# Evaluation of a pneumonia multiplex PCR panel for detection of bacterial respiratory tract pathogens from serial specimens collected from hospitalized COVID-19 patients

**DOI:** 10.1007/s10096-022-04466-9

**Published:** 2022-06-21

**Authors:** Chaitanya Tellapragada, Karin Andersson Ydsten, Anders Ternhag, Christian G. Giske

**Affiliations:** 1 Division of Clinical Microbiology, Department of Laboratory Medicine, Karolinska Institute, Alfred Nobels Allé 8, 14183 Stockholm, Sweden; 2 Department of Infectious Diseases, Karolinska University Hospital, Stockholm, Sweden; 3 Department of Medicine, Solna, Karolinska Institute, Stockholm, Sweden; 4 Department of Clinical Microbiology, Karolinska University Hospital, Stockholm, Sweden

## Abstract

We investigated the concordance between the Unyvero Hospitalized Pneumonia (HPN) application and quantitative culture for detection of bacterial pathogens from serial lower respiratory tract (LRT) specimens collected from the same subject. Comparison of results from HPN application and culture was evaluated using 69 LRT samples from 27 subjects, using two evaluation approaches. False positive detections by the HPN application was 29% (20/69) in Evaluation I vs 10% (7/68) in Evaluation II. Additional pathogens detected by the HPN application could be confirmed in many instances by culture positivity for the same organism from previous or subsequent samples from the same subject.

**Supplementary Information:**

The online version contains supplementary material available at 10.1007/s10096-022-04466-9.

## Introduction

Since the introduction of multiplex PCR-systems for diagnosis of pneumonia, numerous comparative studies to the reference methodology — quantitative respiratory culture — have been performed [1–3]. Results from these studies indicate that the PCR-based diagnostic systems can provide rapid results for both pathogen identification and resistance markers as compared to the microbiological culture-based diagnosis. Furthermore, some of these studies have demonstrated that the PCR-based systems can detect additional pathogens that were not reported by culture. Interpretation of the results with regard to these additional pathogens detected by the PCR-based systems can often be challenging in the absence of culture positivity. Consequently, low positive predictive values (PPVs) of the PCR-based systems, ranging from 46.9 to 78.6% were reported in the published literature, when the comparisons were done assuming that the conventional microbiologic techniques are 100% sensitive and specific [3, 4]. The performance characteristics of the Unyvero Hospitalized Pneumonia (HPN) application (Curetis GmbH, Germany) in comparison with standard-of-care microbiological culture for detection of bacterial pathogens from lower respiratory tract (LRT) samples obtained from critically ill COVID-19 patients were recently reported [3].

Herein, we present a follow-up study aimed to investigate the concordant and discrepant results comparing the Unyvero HPN and culture results for detection of microorganisms from serial specimens collected from the same subject.

## Material and methods

A laboratory-based evaluation study was undertaken at the clinical microbiology laboratory of Karolinska University Hospital (KUH), Solna, Stockholm, Sweden. Frozen lower respiratory tract (LRT) samples from the biobank of the study laboratory were tested with the Unyvero HPN application. All samples had been subjected to standard of care (SoC) testing previously. Detailed description of the methods used for testing the samples using the SoC and the HPN application was described in detail previously [3].

### Clinical samples and data

Samples for the present evaluation were selected from the laboratory information system (LIS) for patients who met the following criteria: (1) samples were obtained from subjects admitted in the intensive care unit with COVID-19, (2) samples were collected from adult patients, (3) at least two or more samples collected on separate days were available from an individual subject, and (4) samples from patients with at least one episode of culture positivity for an HPN panel organism. Sample types, sample collection dates, and microbiology culture findings of the study samples and baseline demographic data of the study subjects were retrospectively collected from the LIS. Clinical data of the study subjects were retrospectively collected from electronic health records by the study physicians.

### Definitions used for interpretation of the HPN application results

#### Full concordance (FC)

Full concordance was considered when the results from the HPN application were identical for the presence/absence of one or more pathogens by SoC: samples with SoC + /HPN + or SoC − /HPN − for a given pathogen; normal microbiota on SoC/HPN − ; only off-panel pathogen SoC + / HPN − .

#### Concordance by correlation (CC)

Concordance by correlation was considered when HPN application was positive for a pathogen that was negative by SoC from the same sample but was positive by culture in a previous or a subsequent sample from the same patient within ± 7 days.

#### Partial concordance (PC)

Partial concordance was considered when HPN application detected the same pathogens that were detected by SoC plus additional pathogen(s) that were not detected by SoC and failed achieving concordance by correlation.

#### Discordance (*D*)

Discordance was considered when an on-panel pathogen was culture positive but was not detected by the HPN application (SoC + /HPN −). Discordant results were also considered when a sample had only one pathogen detected by the HPN application but was not detected by SoC (SoC − /HPN +).

#### Concordance and discordance (*C*/*D*)

There were samples that were positive for more than one pathogen by HPN application but were not detected by SoC. One pathogen was concordant by correlation by culture in a previous or a subsequent sample from the same patient with in ± 7 days; the other pathogen was not detected by SoC.

### Data analysis

The first evaluation (Evaluation I) was performed in a typical manner for a comparative study where Unyvero results were compared with culture results per subject. The second evaluation (Evaluation II) explored interpretation of Unyvero false-positive results which were corroborated by culture from a different sample taken at a later or at an earlier time point from the same subject. In this approach, all Unyvero false positive results were considered true positives, if culture confirmed this result for any other sample (collected at an earlier or later time point) from the same subject.

## Results

### Baseline characteristics of the study samples:

Sixty-nine samples obtained from 27 subjects (15 patients with two, 9 patients with three, and three patients with four samples collected at subsequent time point per subject) were included. Collection of the first respiratory sample for microbiological investigations before administration of an antibiotic was done in 15/27 (55.5%) subjects. Clinical characteristics of the study subjects are listed in Table S1.

### Performance of the HPN application

**Fig. 1:**
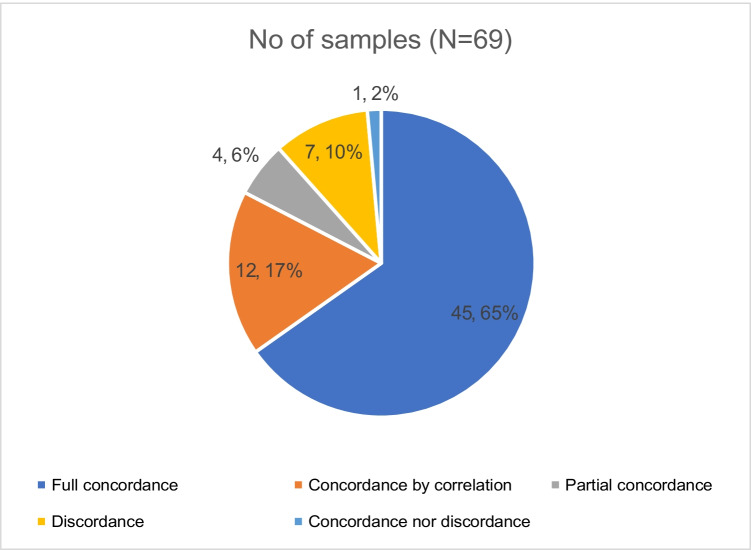
Distribution of the study samples based on their concordance and discordance with SoC results

Full concordance of results from both methods was observed among 45/69 (65.2%) samples (Fig. 1). Nine of the 27 patients with 22 samples (Table S2) showed 100% fully concordant results for all samples collected. Concordance by correlation (CC) was observed among 12/69 (17.3%) samples. At least one episode of CC could be achieved in 11/27 patients (Tables 1 and 2).CC could be achieved from a previous sample and a subsequent sample collected in six subjects each. Discordant results were observed among 7/69 (10%) samples. Among these, SoC + /HPN − result for a pathogen was observed among five samples (Table 2). Among these five samples, the HPN application failed at detecting a pathogen in four samples and misidentified the species name in one sample (Subject 23). Among the four samples with SoC + /HPN − results, the SoC results for three samples were above the lower detection limit of the HPN application, and in one sample (sample 2 from subject 16), the culture positivity was below the detection limit of the HPN application. Discordance, in terms of SoC − /HPN + for additional pathogens was observed among two samples (sample 2 from subject 4; sample 3 from subject 23, both listed in Table 1). There were partial concordant results where the Unyvero application detected additional pathogens in 4/69 samples derived from three patients (Table 1) in addition to the fully matched pathogens. These include two *Staphylococcus aureus* and two *Serratia marcescens*. Finally, concordance and discordance (C/D) was observed in one sample (sample 1 from the subject 27, listed in Table 1).

**Table 1:** Samples from study subjects with additional organisms detected by the Unyvero HPN application

Subject ID	Samples	Days between previous culture	SoC result	Unyvero result	Result interpretation
3	Sample 1	–	*Stenotrophomonas maltophilia* *Serratia marcescens*	*S. maltophilia*; *S. marcescens* *S. aureus*	PC
	Sample 2	7	*S. maltophilia*	*S. maltophilia*; *S. marcescens*	CC^c^
	Sample 3	8	*S. maltophilia*	*S. maltophilia*; *S. marcescens*	PC
	Sample 4	5	*S. maltophilia*	*S. maltophilia*	FC
4	Sample 1	–	*S. aureus*	*S. aureus*	FC
	Sample 2	9	Normal microbiota	*P. aeruginosa*	D^a^
5	Sample 1	–	*S. aureus*	*S. aureus*; *Escherichia coli*	CC^d^
	Sample 2	5	*Morganella morganii*; *E. coli*	*M. morganii*; *E. coli* *S. aureus*	CC^c^
9	Sample 1	–	*S. aureus*; *Klebsiella variicola*	*S. aureus*; *K. variicola* *Klebsiella oxytoca*	FC^b^
	Sample 2	17	*P. aeruginosa*	*P. aeruginosa*; *S. aureus*	PC
10	Sample 1	–	Normal microbiota	*S. aureus*	CC^d^
	Sample 2	7	*S. aureus*	*S. aureus*	FC
12	Sample 1	–	*Streptococcus pneumoniae*	*S. pneumoniae*	FC
	Sample 2	3	*S. aureus*; *Haemophilus influenzae*	*S. aureus*; *H. influenzae* *S. pneumoniae*	CC^c^
	Sample 3	5	Normal microbiota	Negative	FC
14	Sample 1	–	*Candida* spp (off-panel organism)	Negative	FC
	Sample 2	5	*S. marcescens*	*S. marcescens*; *S. aureus*	CC^d^
	Sample 3	6	*S. marcescens*; *S. aureus*	*S. marcescens*; *S. aureus*	FC
19^e^	Sample 1	–	Normal microbiota	*K. oxytoca*	CC^d^
	Sample 2	16	*K. oxytoca*	*K. oxytoca*	FC
	Sample 3	19	Normal microbiota	Negative	FC
21	Sample 1	–	Normal microbiota	Negative	FC
	Sample 2	4	*S. aureus*	*S. aureus*; *S. marcescens*	PC
	Sample 3	10	*S. aureus*	*S. aureus*	FC
23	Sample 1	–	*S. aureus*	*S. aureus*	FC
	Sample 2	1	Normal microbiota	*S. aureus*	CC^c^
	Sample 3	13	Normal microbiota	*S. aureus*	D
25^e^	Sample 1	–	*P. aeruginosa*	*P. aeruginosa*	FC
	Sample 2	10	Normal microbiota	*P. aeruginosa*	CC^c^
26	Sample 1	–	Normal microbiota	*S. aureus*	CC^d^
	Sample 2	5	*S. aureus*	*S. aureus*	FC
27	Sample 1	–	Normal microbiota	*P. aeruginosa*; *S. aureus*	C/D
	Sample 2	3	*S. aureus*	*S. aureus*	FC
	Sample 3	9	Normal microbiota	Negative	FC

*FC* full concordance

^a^*P. aeruginosa* was detected in a urine sample 1 day before

^b^*K. variicola* and *K. oxytoca* probably could not be differentiated on the culture plate due to same colony morphology

^c^Concordance with a previous culture

^d^Concordance with a subsequent culture

^e^Concordance with SoC results of a subsequent sample from the same patient that is not included in the present study

**Table 2:** Study subjects with at least one episode of discordant results

Subject ID	Samples	Days between previous culture	SoC result	Unyvero result	Concordance of the Unyvero result with previous or subsequent culture (Y/N)
6	Sample 1	–	Normal microbiota	Negative	FC
	Sample 2	13	*S. aureus*	Negative	D
11	Sample 1		Normal microbiota	*K. variicola*; *K. oxytoca*	CC**^1^
	Sample 2	3	*K. oxytoca*	Negative	D
16#	Sample 1	–	*S. aureus*	*S. aureus*	FC
	Sample 2	0	*S. aureus*	Negative	D
	Sample 3	8	Normal microbiota	*S. aureus*	CC*
	Sample 4	7	Normal microbiota	Negative	FC
18	Sample 1	–	*S. aureus*	Negative	D
	Sample 2	8	Normal microbiota	Negative	FC
	Sample 3	1	Normal microbiota	Negative	FC
22	Sample 1	–	Normal microbiota	Negative	FC
	Sample 2	7	*Citrobacter koseri*	*Citrobacter freundii*	D

^*^Concordance with a previous culture

^#^Concordance with SoC results of a sample that is not included in the present study, but from the patient chart

^1^* K. variicola* and *K. oxytoca* probably could not be differentiated on the culture plate due to same colony morphology

### Comparison of the results from HPN application with SoC testing

#### Evaluation I

When the results from the HPN application were compared with the culture findings per subject time point, the HPN application generated false positive results in 20/69 (29%) samples (Table 3).

**Table 3:** Results from both methods in 2 × 2 tables as individual samples without correlation with prior or subsequent culture findings

Total (*N* = 69)	SoC Positive (31)	SoC Negative (38)	Total
HPN Positive (47)	27	20*	47
HPN Negative (22)	4	18	22
Total	31	38	69

^*^9 of 20 FP had one additional pathogen by Unyvero and one or more concordant pathogens by both methods

#### Evaluation II

When the results from the HPN application were compared with culture findings per subject time point along with correlation with a previous or a subsequent culture finding, false positive results were observed in 7/68 (10%) samples (Table 4).

**Table 4:** Results from both methods in 2 × 2 tables as paired samples with correlation with prior or subsequent culture findings

	SoC Positive (43)	SoC Negative (25)	Total
HPN Positive (46)	39	7**	46
HPN Negative (22)	4	18	22
Total	31	38	68*

^*^One sample with neither concordance nor discordance results were excluded, thus 68 samples

^**^4 of 7 FP had one additional pathogen by Unyvero and one or more concordant pathogens by both methods

## Discussion

In this study, the false positive results generated from the Unyvero HPN application could be reduced from 29 to 10% by correlating with the culture findings from a previous or a later respiratory sample. Detection of additional pathogens from a given respiratory sample using rapid molecular diagnostic tests has been a consistent observation in several studies when the results from these tests were directly compared with the culture findings [5, 6], and the approach to use a ± 7-day time-window, as in the present study, could be a rational way to validate new diagnostic tests.

At least one additional pathogen was detected from 20/69 (29%) of the samples tested, and the additional pathogens detected in 12/20 (60%) samples were detected by culture from a previous or a subsequent sample collected (± 7 days) and tested from the same subject in six samples each. This observation underscores the ability of the HPN application in detecting a potential pneumonia pathogen earlier than culture and/or very early during infection. Additional pathogens detected from some of the study samples could not be correlated with culture positivity for the same pathogen from previous or subsequent samples, and results from these tests were considered as discordant or partial concordant (Tables 1 and 2). The exact reasons for detection of additional pathogens from these samples could not be determined in the present study. However, it is possible that some of the microorganisms detected were colonizers present in low amounts below the current diagnostic thresholds for reporting. Another important factor that could have had led to additional detection (SoC − / HPN +) for a given organism is the antibiotic exposure before sample collection. The Unyvero HPN application detected an additional pathogen from at least one sample collected from 14/27 (52%) subjects in the present study. Among these 14 subjects, 10 of them had exposure to antibiotics before the collection of samples for culture, and it is plausible that the exposure of the antibiotic had a negative influence on the yield of the cultures from these samples.

In conclusion, the additional pathogens detected by the Unyvero HPN application from a given LRT sample could be confirmed in many instances by culture positivity for the same microorganism from a previous or a subsequent sample obtained from the same subject.

## Supplementary Information

Below is the link to the electronic supplementary material.

Supplementary file1 (DOCX 27 KB)

## Data Availability

All relevant data are available in the manuscript.
