# Supplementary material for: Evaluation of a pneumonia multiplex PCR panel for detection of bacterial respiratory tract pathogens from serial specimens collected from hospitalized COVID-19 patients
Source: Eur J Clin Microbiol Infect Dis. 2022 Jun 21;41(7):1093–8. doi: 10.1007/s10096-022-04466-9 (PMC9210330; doi:10.1007/s10096-022-04466-9)
Supplement: Supplementary file 1 — Supplementary file1 (DOCX 27 KB) [file 10096_2022_4466_MOESM1_ESM.docx]

| **Subject ID**  **Table S1: Characteristics of the study subjects.** | **Age** | **Gender** | **Days between previous culture** | **Sample Type** | **LoS- Hospital**  **(in days)** | **LoS-ICU (in days)** | **Hospital mortality** | **Antibiotics* before sample collection** | **Antibiotics* after culture report** |
| --- | --- | --- | --- | --- | --- | --- | --- | --- | --- |
| **1** | 73 | M | -  11 | Tracheal secretion  Tracheal secretion | 17 | 14 | Yes | Cefotaxime  Cefotaxime | Cefotaxime  Pip/tazobactam |
| **2** | 70 | M | -  2  4  3 | Tracheal secretion  Tracheal secretion  Tracheal secretion  Tracheal secretion | 50 | 29 | No | None  None  None  None | None  None  None  Meropenem |
| **3** | 65 | M | -  7  8  5 | Tracheal secretion  Tracheal secretion  Tracheal secretion  Tracheal secretion | 66 | 35 | No | Meropenem  Meropenem + moxifloxacin  Meropenem + TMP/SMX  None | Meropenem + moxifloxacin  Meropenem + TMP/SMX  TMP/SMX  TMP/SMX |
| **4** | 64 | M | -  9 | Tracheal secretion  Tracheal secretion | 46 | 34 | No | Cefotaxime  Pip/tazobactam + cloxacillin | Pip/tazobactam  Meropenem |
| **5** | 57 | M | -  5 | Tracheal secretion  Tracheal secretion | 47 | 32 | No | Cefotaxime  Cefotaxime | Cefotaxime  Pip/tazobactam |
| **6** | 57 | M | -  13 | Tracheal secretion  Tracheal secretion | 82 | 48 | No | None  Meropenem | None  Ciprofloxacin + Pip/tazobactam |
| **7** | 53 | M | -  8 | Tracheal secretion  Tracheal secretion | 37 | 29 | No | None  Cefotaxime | Cefotaxime  Cefotaxime (later Pip/tazobactam) |
| **8** | 47 | M | -  7  1 | Tracheal secretion  Tracheal secretion  Tracheal secretion | 29 | 19 | No | Pip/tazobactam  None  Meropenem | Pip/tazobactam  Meropenem  Meropenem |
| **9** | 41 | M | -  17 | Tracheal secretion  Tracheal secretion | 38 | 29 | No | None  None | None  None |
| **10** | 40 | M | -  8 | Tracheal secretion  Tracheal secretion |  |  | Not available | None  None | None  Meropenem |
| **11** | 39 | F | -  3 | Tracheal secretion  Tracheal secretion | 11 | 10 | Yes | Cefotaxime  Meropenem | Meropenem  Meropenem |
| **12** | 52 | M | -  3  5 | PSB  BAL  BAL | 29 | 14 | No | None  Benzylpenicillin  Meropenem + vancomycin + clindamycin | None  Meropenem + vancomycin  Meropenem + vancomycin + clindamycin |
| **13** | 51 | M | -  5 | BAL  Bronchialsecretion | 68 | 44 | No | Pip/tazobactam + linezolid  Linezolid | Pip/tazobactam + linezolid  Linezolid |
| **14** | 40 | M | -  5  6 | BAL  Tracheal secretion  Sputum | 61 | 46 | No | Cefotaxime  None  None | Cefotaxime  None  None |
| **15** | 39 | M | -  3  5 | PSB  PSB  BAL | 123 | 82 | No | None  Cefotaxime  Meropenem + vancomycin + clindamycin | Cefotaxime  Meropenem + vancomycin + clindamycin  Meropenem + vancomycin + clindamycin |
| **16** | 38 | M | -  0  8  7 | BAL  PSB  Tracheal secretion  BAL | 33 | 33 | Yes | None  None  Cefotaxime  None | None  Meropenem  Cefotaxime  None |
| **17** | 71 | M | -  4 | Tracheal secretion  Tracheal secretion | 21 | 18 | Yes | None  Cefotaxime | Cefotaxime  Pip/tazobactam |
| **18** | 65 | M | -  8  1 | BAL  BAL  Tracheal secretion | 109 | 83 | No | None  None  None | Pip/tazobactam  None  None |
| **19** | 71 | F | -  16  19 | Tracheal secretion  Tracheal secretion  Tracheal secretion | 64 | 64 | Yes | None  None  Imipenem+ TMP/SMX | None  None  Imipenem+ TMP/SMX |
| **20** | 66 | M | -  3 | Tracheal secretion  Tracheal secretion | 53 | 36 | No | None  TMP/SMX | None  Pip/tazobactam |
| **21** | 66 | F | -  4  10 | BAL  BAL  Tracheal secretion | 42 | 21 | No | Pip/tazobactam  None  Meropenem | Pip/tazobactam  None  Meropenem |
| **22** | 64 | M | -  7 | Tracheal secretion  Tracheal secretion | 70 | 54 | No | None  Pip/tazobactam | None  Pip/tazobactam |
| **23** | 62 | M | -  1  13 | Tracheal secretion  Tracheal secretion  Tracheal secretion | 17 | 17 | Yes | None  Cefotaxime  Pip/tazobactam | Cefotaxime  Cefotaxime + linezolid  Pip/tazobactam |
| **24** | 60 | M | -  20 | Tracheal secretion  Tracheal secretion | 44 | 42 | Yes | Pip/tazobactam + vancomycin  None | Cefotaxime + vancomycin  None |
| **25** | 59 | F | -  10 | Tracheal secretion  Tracheal secretion | 57 | 56 | Yes | Levofloxacin + vancomycin + Pip/tazobactam  Ceftolozane/tazobactam + Colistin | Levofloxacin +vancomycin + pip/tazobactam + TMP/SMX  Ceftolozane/tazobactam + Colistin |
| **26** | 51 | M | -  5 | Tracheal secretion  Tracheal secretion | 35 | 22 | No | None  None | Meropenem  None |
| **27** | 55 | M | -  3  9 | BAL  BAL  Tracheal secretion | 34 | 27 | No | Cefotaxime  Cefotaxime  None | Cefotaxime  Pip/tazobactam  None |

*Only antibacterial agents are listed here.

LoS, length of stay; BAL, bronchoalveolar lavage; PSB, protected specimen brush; Pip/tazobactam, piperacillin/tazobactam; TMP/SMX, trimethoprim-sulfamethoxazole.

**Table S2: Samples with full concordance in all episodes**

| Subject ID | Samples | Days between previous Culture | SoC result | Unyvero result | Result interpretation |
| --- | --- | --- | --- | --- | --- |
| 1 | Sample 1 | - | Normal microbiota | Negative | FC |
|  | Sample 2 | 11 | *Escherichia coli* | *E. coli* | FC |
| 2 | Sample 1 | - | Normal microbiota | Negative | FC |
|  | Sample 2 | 2 | Normal microbiota | Negative | FC |
|  | Sample 3 | 4 | *Pseudomonas aeruginosa* | *P. aeruginosa* | FC |
|  | Sample 4 | 3 | *P. aeruginosa* | *P. aeruginosa* | FC |
| 7 | Sample 1 | *-* | *Staphylococcus aureus* | *S. aureus* | FC |
|  | Sample 2 | 8 | *S. aureus* | *S. aureus* | FC |
| 8 | Sample 1 | - | Normal microbiota | Negative | FC |
|  | Sample 2 | 7 | *S. aureus* | *S. aureus* | FC |
|  | Sample 3 | 1 | *S. aureus* | *S. aureus* | FC |
| 13 | Sample 1 | - | Normal microbiota | Negative | FC |
|  | Sample 2 | 5 | *S. aureus* | *S. aureus* | FC |
| 15 | Sample 1 | - | Normal microbiota | Negative | FC |
|  | Sample 2 | 3 | *Enterobacter cloacae* | *E. cloacae* complex | FC |
|  | Sample 3 | 5 | Normal microbiota | Negative | FC |
| 17 | Sample 1 | - | Normal microbiota | Negative | FC |
|  | Sample 2 | 4 | *E. cloacae* | *E. cloacae* complex | FC |
| 20 | Sample 1 | - | *Klebsiella pneumoniae* | *K. pneumoniae* | FC |
|  | Sample 2 | 3 | *K. pneumoniae* | *K. pneumoniae* | FC |
| 24 | Sample 1 | - | *S. aureus* | *S. aureus* | FC |
|  | Sample 2 | 20 | *S. aureus* | *S. aureus* | FC |

FC, Full concordance.
